# PGC1α Cooperates with FOXA1 to Regulate Epithelial Mesenchymal Transition through the TCF4-TWIST1

**DOI:** 10.3390/ijms23158247

**Published:** 2022-07-26

**Authors:** Xue-Quan Fang, Mingyu Lee, Woo-Jin Lim, Seonghoon Lee, Chang-Hoon Lim, Ji-Hong Lim

**Affiliations:** 1 Department of Biomedical Chemistry, College of Biomedical & Health Science, Konkuk University, Chungju 380-701, Korea; gkrrnjs654852@kku.ac.kr (X.-Q.F.); lwj0908@kku.ac.kr (W.-J.L.); samron7@kku.ac.kr (S.L.); lchoo1196@kku.ac.kr (C.-H.L.); 2 Department of Applied Life Science, Graduate School, BK21 Program, Konkuk University, Chungju 380-701, Korea; 3 Division of Allergy and Clinical Immunology, Department of Medicine and Brigham and Women’s Hospital, Harvard Medical School, Boston, MA 02115, USA; leemk08@gmail.com; 4 Center for Metabolic Diseases, Konkuk University, Chungju 380-701, Korea

**Keywords:** EMT, lung cancer, PGC1α, FOXA1, ID1, TCF4, TWIST1

## Abstract

The peroxisome proliferator-activated receptor gamma coactivator 1-alpha (PGC1α) is a critical transcriptional coactivator that maintains metabolic homeostasis and energy expenditure by cooperating with various transcription factors. Recent studies have shown that PGC1α deficiency promotes lung cancer metastasis to the bone through activation of TCF4 and TWIST1-mediated epithelial–mesenchymal transition (EMT), which is suppressed by the inhibitor of DNA binding 1 (ID1); however, it is not clear which transcription factor participates in PGC1α-mediated EMT and lung cancer metastasis. Here, we identified forkhead box A1 (FOXA1) as a potential transcription factor that coordinates with PGC1α and ID1 for EMT gene expression using transcriptome analysis. Cooperation between FOXA1 and PGC1α inhibits promoter occupancy of TCF4 and TWIST1 on CDH1 and CDH2 proximal promoter regions due to increased ID1, consequently regulating the expression of EMT-related genes such as CDH1, CDH2, VIM, and PTHLH. Transforming growth factor beta 1 (TGFβ1), a major EMT-promoting factor, was found to decrease ID1 due to the suppression of FOXA1 and PGC1α. In addition, ectopic expression of ID1, FOXA1, and PGC1α reversed TGFβ1-induced EMT gene expression. Our findings suggest that FOXA1- and PGC1α-mediated ID1 expression involves EMT by suppressing TCF4 and TWIST1 in response to TGFβ1. Taken together, this transcriptional framework is a promising molecular target for the development of therapeutic strategies for lung cancer metastasis.

## 1. Introduction

Lung cancer is the most dangerous solid cancer and can exhibit distant metastasis to the brain, liver, bones, and adrenal glands; metastatic bone tumors are observed in approximately 30–40% of patients with advanced lung cancer [1,2]. It is clear that the epithelial–mesenchymal transition (EMT), which is activated mainly by transforming growth factor β1 (TGFβ1) derived from cancer-associated fibroblasts (CAFs) and tumor-associated macrophages (TAMs), promotes the spread of epithelial cancer cells to other organs [3]. EMT is characterized by downregulation of E-cadherin (epithelial marker) and upregulation of N-cadherin and vimentin (mesenchymal markers), which are transcriptionally regulated by several types of transcription factors, such as SNAIL1, SNAIL2, ZEB1, ZEB2, and TWIST1 [4]. Recent reports have demonstrated that the E-box binding protein, TCF4 (also called ITF-2 or E2-2), interacts and cooperates with TWIST1 to regulate lineage-specific differentiation in mesoderm and neural crest stem cells [5] and EMT in epithelial lung cancer cells [2]. In addition, ID1 (inhibitor of DNA binding 1), an inhibitor of the basic helix-loop-helix (bHLH) transcription factor, inhibits EMT by disrupting the interaction between TCF4 (E-box binding protein) and TWIST1 (bHLH transcription factor) during EMT in lung epithelial cancer cells [2]. However, it is not fully understood which stimuli and signaling cascades fine-tune TCF4- and TWIST1-mediated EMT in lung cancer.

The peroxisome proliferator-activated receptor gamma coactivator 1-alpha (PGC1α) is a transcriptional coactivator that regulates glucose and lipid metabolism and energy consumption in the liver, adipose, and skeletal muscle [6]. Mechanistically, PGC1α interacts with various nuclear factors and transcription factors, including peroxisome proliferator-activated receptors (PPARs), thyroid hormone receptors (THRs), retinoid receptors (RARs), glucocorticoid receptor (GR), estrogen receptor (ER), hepatic nuclear factor-4 (HNF-4), liver X receptor (LXR), estrogen-related receptors (ERRs), nuclear respiratory factor 1 (NRF-1), nuclear respiratory factor 2 (NRF-2), forkhead box O1 (FOXO1), and sterol regulatory element-binding protein-1 (SREBP-1) to boost target gene expression and subsequently maintain metabolic homeostasis [7]. In addition to the functional role of PGC1α in energy metabolism, several reports have shown that PGC1α promotes or suppresses tumor initiation, growth, and metastasis in malignant melanoma and liver, breast, lung, and prostate cancer [8,9,10,11,12]. Given that PGC1α and its interacting transcription factors regulate energy metabolism, several reports have shown that PGC1α regulates cancer growth and metastasis through ERRα-mediated mitochondrial oxidative metabolism in malignant melanoma and prostate cancer [12,13,14]. Loss of PGC1α was found to promote lung cancer metastasis through transcriptional circuits regulating EMT-related signaling events in a genetically engineered mouse model with deletion of a single allele of ppargc1a and overexpression of Kras^G12V^ [2]. Although a few reports have shown that PGC1α acts as an essential component of the transcriptional circuit to regulate tumor growth, EMT, and metastasis in lung cancer [2,13,14,15], the pivotal transcription factor that cooperates with PGC1α to regulate tumor behavior in lung cancer has not been identified.

Forkhead box protein A1 (FOXA1, also known as hepatocyte nuclear factor (HNF) 3A), is a transcription factor that is expressed in various tissues such as the liver, breast, pancreas, bladder, prostate, colon, and lung, and is involved in embryonic development and tissue-specific gene expression. FOXA1 defines cancer specificity and regulates EMT in breast, prostate, and lung cancers [16,17,18]. Numerous reports have revealed that FOXA1 suppresses EMT and cancer metastasis in lung, breast, prostate, gastric, and pancreatic cancers [16,17,18,19,20,21]. However, the precise molecular mechanism by which FOXA1 regulates EMT in lung cancer has not been fully elucidated.

## 2. Results

### 2.1. PGC1α Is a Potential Transcriptional Coactivator of FOXA1

**Figure 1:**
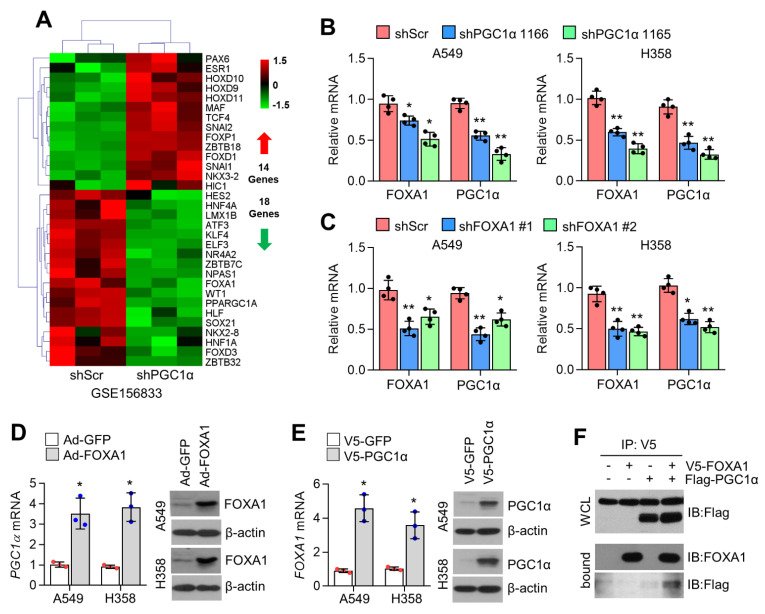
PGC1α associates with FOXA1. (**A**) Heat map for the 14 upregulated and 18 downregulated transcription factors encoding gene expression of the RNA seq analysis in control or PGC1α knock-down A549 cells. (**B**,**C**) FOXA1 and PGC1α mRNA expression in (**B**) PGC1α and (**C**) FOXA1 silenced A549 and H358 lung cancer cells. (**D**,**E**) PGC1α and FOXA1 mRNA expression in (**D**) FOXA1 and (**E**) PGC1α overexpressed A549 and H358 cells. Overexpressed FOXA1 and PGC1α were shown. (**F**) Interaction of FOXA1 and PGC1α. Values represent mean ± SD (n = 3 or 4). * *p* < 0.05 and ** *p* < 0.01 by unpaired Student’s *t*−test for two experimental comparison and one-way ANOVA with Tukey post-test for multiple comparison.

We previously demonstrated that PGC1α, which regulates metabolic homeostasis by cooperating with various transcription factors such as NRF, PPAR, and ERR, suppresses EMT by increasing ID1 in lung epithelial cancer cells [2]. However, a transcription factor that cooperates with PGC1α, which regulates ID1 and EMT in lung cancer cells, has not been identified. Because it is well-known that PGC1α and its interacting transcription factors express their own genes via an autoregulatory feedback loop, among transcription factors that were decreased in response to PGC1α suppression, we tried to identify the transcription factor that regulates ID1 and EMT by cooperating with PGC1α. Thus, we recalled previous RNA-seq data (GSE156833) and analyzed the mRNA levels of up- and downregulated transcription factors in PGC1α knockdown A549 cells [2]. Interestingly, 14 upregulated transcription factor genes, such as EMT-promoting SNAI1, SNAI2, and TCF4 were observed (Figure 1A). Eighteen downregulated genes, including HNF4A, NR4A2, and KLF4, which are known to interact with PGC1α, were observed (Figure 1A) [22,23,24]. Increased observations have revealed that FOXA1 (also known as HNF3A) is associated with EMT in multiple types of cancer, such as prostate [17,25], breast [16], pancreatic [20], and lung cancer [21]. Thus, we speculate that cooperation between FOXA1 and PGC1α may participate in EMT-related gene expression by regulating ID1 expression in lung cancer cells. Consistent with the transcriptome analysis (Figure 1A), decreased FOXA1 mRNA levels were observed in PGC1α-silenced A549 and H358 lung cancer cells (Figure 1B). Figure 1C shows that PGC1α mRNA levels were downregulated in FOXA1 knockdown A549 and H358 cells. Similarly, PGC1α transcript levels were significantly increased in adenoviral FOXA1-transduced cells (Figure 1D). Figure 1E shows that overexpression of PGC1α largely increased FOXA1 expression in A549 and H358 lung cancer cells. In addition, ectopically expressed PGC1α interacted with FOXA1 (Figure 1F). These results indicate that PGC1α and FOXA1 cooperate with each other to regulate gene expression in lung cancer cells.

### 2.2. PGC1α Cooperates with FOXA1 to Regulate ID1 Expression

**Figure 2:**
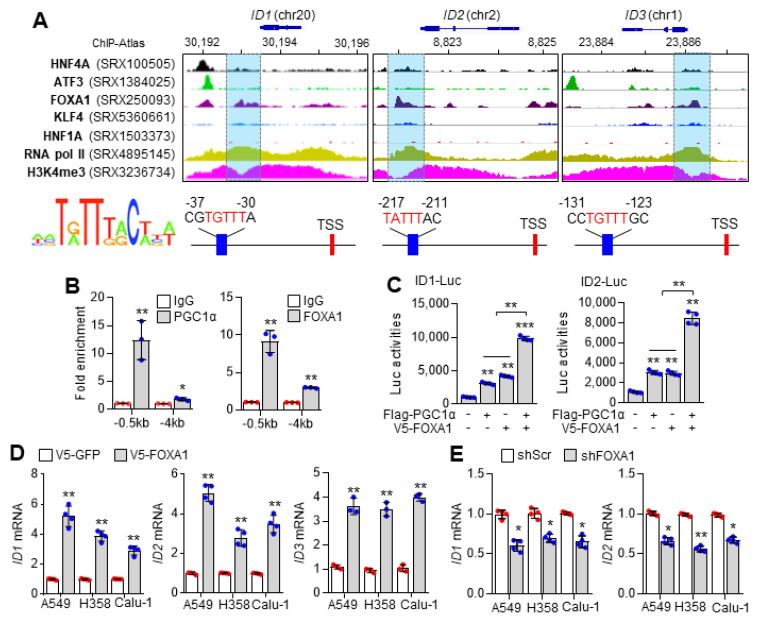

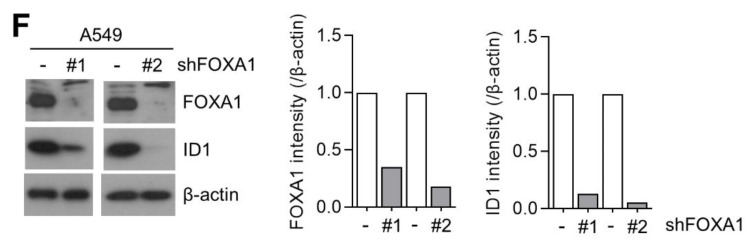
Cooperation between of PGC1α and FOXA1 regulates ID1 expression. (**A**) ChIP seq profiles of HNF4A, ATF3, FOXA1, KLF4, HNF1A, RNA pol II, and H3K4−me3 indicating transcriptionally active region of ID1, ID2, and ID3. Representative images obtained by ChIP-Atlas (https://chip-atlas.org/ (accessed on 10 September 2021)) and integrative genomic viewer (IGV). FOXA1 consensus binding sequences of ID1, ID2, and ID3 are represented. (**B**) ChIP for PGC1α, FOXA1, and IgG, followed by ChIP−qPCR at −0.5 and −4 kb of ID1 promoter loci in A549 cells. (**C**) ID1 and ID2 promoter luciferase analysis. (**D**) Expression of the ID1, ID2, and ID3 genes in FOXA1 overexpressed A549, H358, and Calu−1 cells quantified by qPCR. (**E**) Expression of the ID1 and ID2 genes upon FOXA1 knockdown. (**F**) Western blots of FOXA1 and ID1 in the control and FOXA1 silenced A549 cells. Quantitative FOXA1 and ID1 levels represented (right panel). Values represent mean ± SD (n = 3 or 4). * *p* < 0.05, ** *p* < 0.01, and *** *p* < 0.001 by unpaired Student’s *t*−test for two experimental comparisons and one-way ANOVA with Tukey post-test for multiple comparisons.

Oh et al. showed that PGC1α transcriptionally activates ID1, ID2, and ID3, but not ID4 expression in lung cancer cells [2]. However, the precise mechanism by which PGC1α, as a transcriptional coactivator, enhances ID transcription has not been identified. Thus, we analyzed the promoter sequences of ID1, ID2, and ID3 and identified which transcription factor downregulated by PGC1α silencing (Figure 1A) interacts with the transcriptionally active ID promoter. Interestingly, similar nucleotide sequences to FOXA1 (HNF3A) consensus binding site (T[A/G]TT[G/T]AC) [26] and strong promoter occupancy of FOXA1 with RNA pol II and H3K4-me3 on the proximal promoter region of ID1, ID2, and ID3 were observed using the publicly available ChIP-seq database (Figure 2A). Indeed, stronger binding of PGC1α and FOXA1 to the ID1 promoter at −0.5 kb rather than at −4 kb from the transcription start site (TSS) was observed (Figure 2B). To investigate whether PGC1α is a potential transcriptional coactivator of FOXA1, the transcriptional activity of FOXA1 was measured in the absence or presence of PGC1α through ID1- and ID2-promoter luciferase assays. Here, increased ID1 and ID2 promoter luciferase activities were observed in the coexpression of PGC1α and FOXA1 compared to PGC1α or FOXA1 alone (Figure 2C). Ectopic expression of FOXA1 increased ID1, ID2, and ID3 mRNA in A549, H358, and Calu-1 lung cancer cells (Figure 2D). In addition, FOXA1 knockdown decreased ID1 and ID2 mRNA (Figure 2E) and protein levels (Figure 2F) in lung cancer cells. These results suggest that FOXA1 acts as a key transcription factor for PGC1α-mediated ID expression in lung cancer cells.

### 2.3. PGC1α and FOXA1 Negatively Regulate TGFβ1-Induced EMT

**Figure 3:**
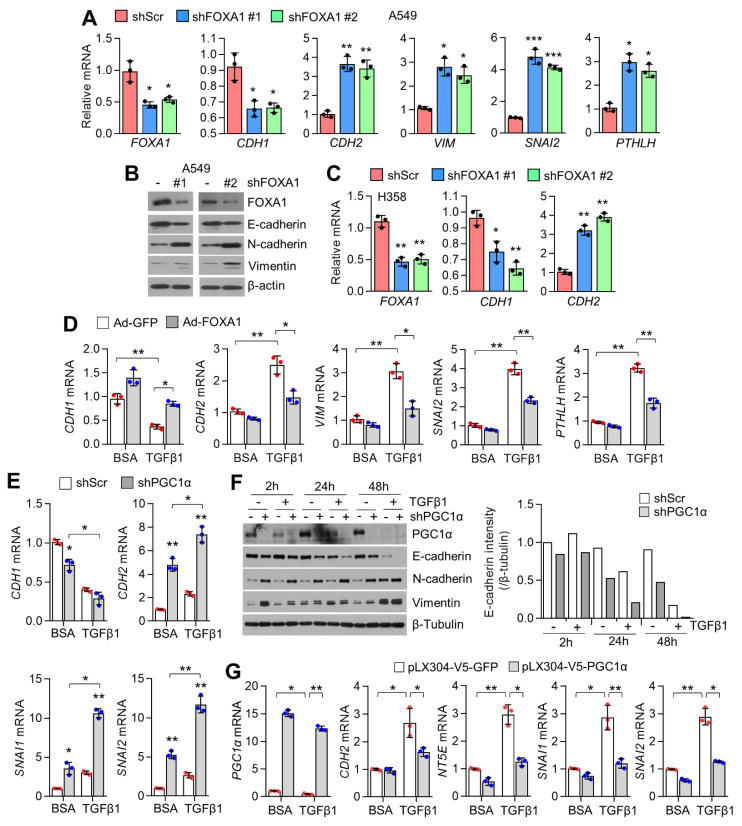
PGC1α and FOXA1 suppress EMT-associated gene expression. (**A**) EMT−related genes and (**B**) proteins expression in FOXA1 silenced A549 cells. (**C**) EMT gene expression in FOXA1 silenced H358 cells. (**D**) Alteration of EMT genes by ectopic GFP or FOXA1 expression upon TGFβ1. A549 cells were infected with adenovirus (1000 pfu/cell) expressing GFP or FOXA1 and incubated for 48 h. After adenoviral transduction, cells were incubated with TGFβ1 (20 ng/mL) for 24 h. (**E**) EMT-associated genes expression in PGC1α silenced A549 cells upon TGFβ1 (20 ng/mL for 24 h). Upper (CDH1 and CDH2) and bottom (SNAI1 and SNAI2). (**F**) EMT-associated proteins expression in PGC1α silenced A549 cells upon TGFβ1 (20 ng/mL). Quantitative E−cadherin levels represented (right panel). (**G**) Changes of EMT−related gene in ectopic PGC1α expression upon TGFβ1. A549 cells were infected with PGC1α expressing lentivirus (1000 pfu/cell) and incubated for 48 h. After lentiviral transduction, cells were incubated with TGFβ1 (20 ng/mL) for 24 h. Values represent mean ± SD (n = 3). * *p* < 0.05, ** *p* < 0.01, and *** *p* < 0.001 by one-way ANOVA with Tukey post-test for multiple comparisons.

Because loss of PGC1α was found to promote lung cancer metastasis to the bone by activating EMT in Kras^G12V^-driven lung cancer models as well as lung adenocarcinoma cells [2], we investigated whether FOXA1, a PGC1α interacting transcription factor, is involved in EMT in lung cancer cells. Here, we found that FOXA1 knockdown decreased CDH1 (epithelial marker) but increased CDH2, VIM, SNAI2, and PTHLH (mesenchymal markers) in A549 lung cancer cells (Figure 3A,B). Similarly, decreased CDH1 and increased CDH2 were observed in FOXA1-silenced H358 lung cancer cells (Figure 3C). In addition, ectopic FOXA1 expression significantly reversed TGFβ1-mediated expression of EMT markers, such as CDH1, CDH2, VIM, SNAI2, and PTHLH, in A549 lung cancer cells (Figure 3D). Moreover, enhanced expression of EMT-related genes (Figure 3E) and proteins (Figure 3F) in the presence of TGFβ1 was observed in PGC1α-silenced A549 lung cancer cells. Ectopically overexpressed PGC1α was found to reverse the EMT gene expression in response to TGFβ1 (Figure 3G). These results demonstrate that cooperation between FOXA1 and PGC1α acts as a negative regulator of TGFβ1-mediated EMT gene expression. PGC1α is required for the induction of various ROS-detoxifying enzymes, including GPx1 and SOD2 [27]. Reactive oxygen species (ROS) are proposed to be involved in tumor metastasis via complicated processes including EMT, migration, invasion and angiogenesis [28]. Thus, we investigated whether PGC1α and FOXA1 participate the induction of ROS-detoxifying enzymes and the maintenance of redox homeostasis in A549 lung adenocarcinoma cells. Decrease SOD2 but not GPx1 were observed in PGC1α-silenced A549 cells (Supplementary Figure S1A). Inversely, FOXA1 silencing significantly increased SOD2 (Supplementary Figure S1B). In addition, reduced intracellular ROS levels were observed in PGC1α and FOXA1-silenced A549 cells (Supplementary Figure S1C). These results reveal that intracellular ROS is not required for EMT controlled by cooperation of PGC1α-FOXA1 in A549 lung adenocarcinoma cells. Increased TGFβ1 signaling-associated downstream target genes such as *TGFB1* and *INHBA* were observed in PGC1α silenced melanoma and lung adenocarcinoma cells [2,15]. Consistent with previous observation, *TGFB1*, a well-known TGFβ1 signaling target gene, was significantly increased in FOXA1 silenced A549 cells (Supplementary Figure S2A,B), indicating that FOXA1 cooperates with PGC1α on the regulation of TGFβ1 signaling target-gene expression.

### 2.4. Inhibition of FOXA1 Promotes EMT by Increasing TCF4-TWIST1 Interactions Due to the Suppression of ID1

**Figure 4:**
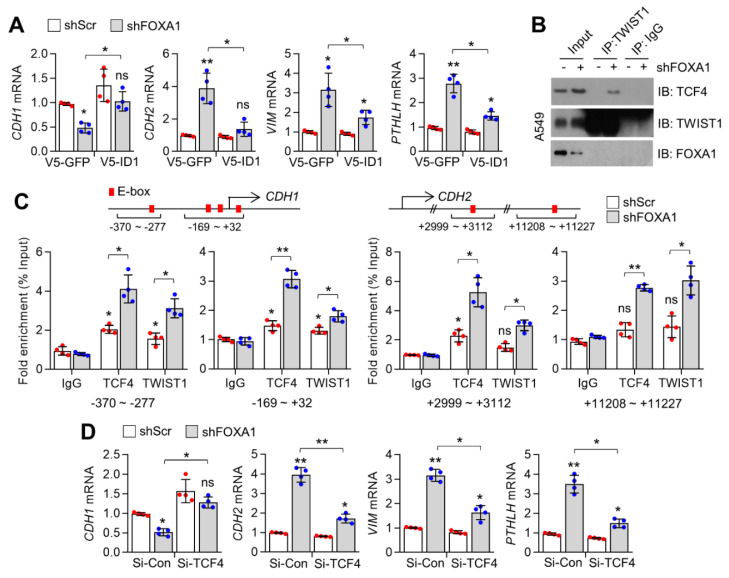
Suppression of TCF4 and TWIST1 by ID1 is linked to loss of FOXA1-induced EMT. (**A**) Changes of EMT−associated genes by FOXA1 silencing in ectopic GFP or ID1 expressing A549 cells. (**B**) Interaction between TCF4 and TWIST1 in FOXA1 knockdown A549 cells. (**C**) Promoter occupancy of TCF4 and TWIST1 on CDH1 and CDH2 promoter in FOXA1 silenced A549 cells. (**D**) Changes of EMT-associated genes by FOXA1 silencing in control or TCF4 knockdown A549 cells. Values represent mean ± SD (n = 4). * *p* < 0.05 and ** *p* < 0.01 by one-way ANOVA with Tukey post-test for multiple comparisons. Not significate ns is normally this meaning.

E-box binding protein TCF4 (ITF-2 or E2-2) promotes EMT by activating the transcriptional activity of TWIST1, and cooperation between TCF4 and TWIST1 in EMT gene expression is tightly controlled by ID1 [2]. Because FOXA1 and PGC1α were found to regulate ID1, ID2, and ID3 in lung cancer cells, we investigated the mechanistic impact of ID1 on the loss of FOXA1-mediated EMT in lung cancer cells. Interestingly, reduction of CDH1 and induction of CDH2, VIM, and PTHLH by loss of FOXA1 was diminished in stable ID1 overexpression (Figure 4A), indicating that ID1 is mechanistically associated with alterations in EMT-related gene expression by FOXA1. In addition, Figure 4B shows that FOXA1 knockdown promoted the interaction of TCF4 and TWIST1. Consistent with this, suppression of ID1 increased promoter occupancy of TCF4 and TWIST1 on E-box containing CDH1 and CDH2 promoter loci [2], and increased TCF4 and TWIST1 were observed on CDH1 and CDH2 promoters in FOXA1 knockdown A549 lung cancer cells (Figure 4C). Moreover, inhibition of TCF4 reversed the EMT gene expression in FOXA1-silenced A549 cells (Figure 4D). These results indicate that the functional role of ID1 in the negative regulation of TCF4 and TWIST1 is closely linked to the loss-of-FOXA1-induced EMT in lung cancer cells.

### 2.5. TGFβ1 Attenuates FOXA1 and PGC1α, Resulting in the Suppression of ID1

**Figure 5:**
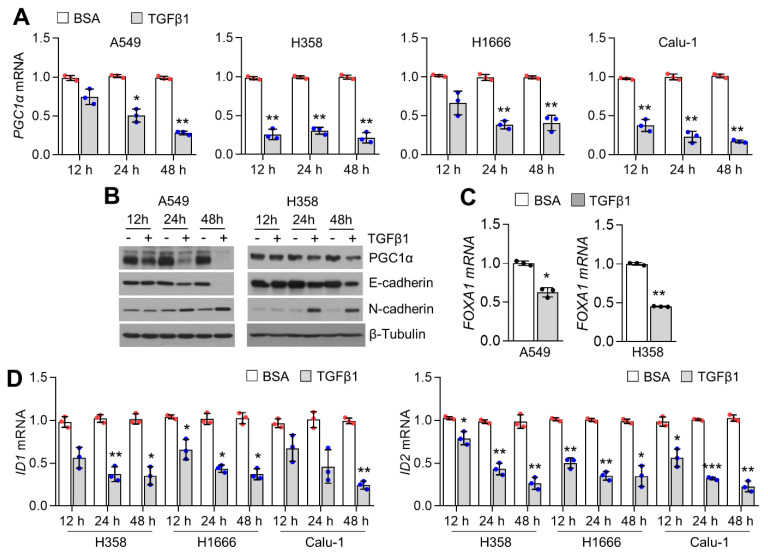

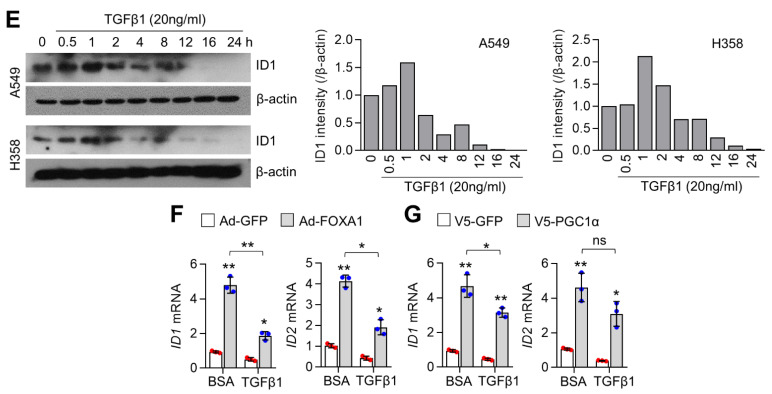
TGFβ1 decreases ID1 by suppressing FOXA1 and PGC1α. (**A**) PGC1α mRNA upon TGFβ1 (20 ng/mL) in a time−dependent manner. PGC1α mRNA levels in TGFβ1−treated cells were compared to BSA−treated cells at indicated time points (12 h, 24 h or 48 h), respectively. (**B**) PGC1α protein expression upon TGFβ1 (20 ng/mL) in a time-dependent manner. (**C**) FOXA1 mRNA in the absence or presence of TGFβ1 (20 ng/mL for 24 h). (**D**) ID1 and ID2 mRNA expression upon TGFβ1 (20 ng/mL) in a time-dependent manner. ID1 and ID2 mRNA levels in TGFβ1−treated cells were compared to BSA−treated cells at indicated time points (12 h, 24 h, or 48 h), respectively. (**E**) ID1 protein in TGFβ1−treated A549 and H358 cells in a time−dependent manner. Quantitative ID1 protein level represented (right panel). (**F**) Changes of ID1 and ID2 mRNA levels by TGFβ1 (20 ng/mL for 24 h) in ectopic FOXA1 or (**G**) PGC1α expressing A549 cells. Values represent mean ± SD (n = 3). * *p* < 0.05, ** *p* < 0.01, and *** *p* < 0.001 by unpaired Student’s *t*−test for two experimental comparisons and one-way ANOVA with Tukey post-test for multiple comparisons.

To understand the physiological relevance related to how decreased PGC1α, FOXA1, and ID1 promote metastasis via EMT in lung cancer, we speculated that TGFβ1, as a major EMT stimulus, could affect the suppression of PGC1α, FOXA1, or ID1. Initially, we measured PGC1α in the absence or presence of TGFβ1, and decreased PGC1α mRNA was observed in TGFβ1-treated A549, H358, H1666, and Calu-1 lung cancer cells (Figure 5A). PGC1α protein and EMT markers (E-cadherin and N-cadherin) were also decreased in TGFβ1-treated A549 and H358 cells (Figure 5B). Because FOXA1 is decreased in PGC1α knockdown cells, we investigated whether TGFβ1 decreased FOXA1 as well as PGC1α. Figure 5C shows that TGFβ1 significantly reduced FOXA1 expression in A549 and H358 lung cancer cells. In addition, ID1 and ID2, which are regulated by PGC1α and FOXA1, were attenuated by TGFβ1 treatment in H358, H1666, and Calu-1 cells in a time-dependent manner (Figure 5D). Decreased ID1 protein levels were found in TGFβ1-treated A549 and H358 cells (Figure 5E). To determine whether FOXA1 and PGC1α are necessary for ID1 and ID2 suppression by TGFβ1, ID1, and ID2 mRNA levels in the absence or presence of TGFβ1, FOXA1- or PGC1α-overexpressing A549 cells were analyzed. Here, we found that decreased ID1 and ID2 by TGFβ1 treatment was reversed in FOXA1- and PGC1α-overexpressing cells compared to control cells (Figure 5F,G). Overall, these results revealed that the role of cooperation between PGC1α and FOXA1 on the transcription of ID1 is functionally linked to TGFβ1-mediated EMT in lung cancer cells.

### 2.6. TGFβ1 Increases TCF4-TWIST1 Interaction by Suppressing ID1, Consequently Activating EMT

**Figure 6:**
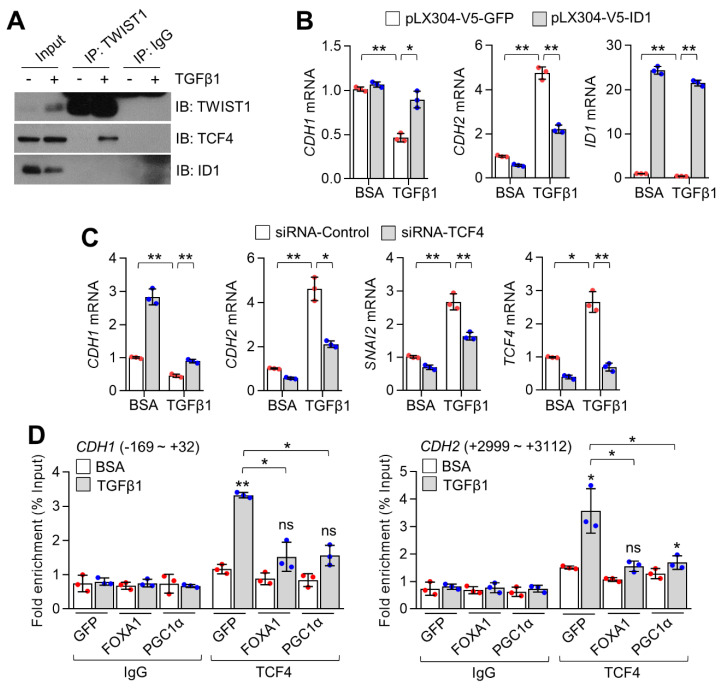
Suppression of TCF4 and TWIST1 by ID1 involves TGFβ1−mediated EMT gene expression. (**A**) Interaction of TCF4 and TWIST1 in the absence or presence of TGFβ1 (20 ng/mL for 24 h). (**B**) EMT-associated gene expression upon TGFβ1 (20 ng/mL for 24 h) in GFP or ID1 stably expressing A549 cells. (**C**) TGFβ1−mediated EMT gene expression in control or TCF4 silenced A549 cells. (**D**) Promoter occupancy of TCF4 on CDH1 and CDH2 promoter in GFP, FOXA1 or PGC1α stably expressing A549 cells. Values represent mean ± SD (n = 3). * p < 0.05 and ** p < 0.01 by one-way ANOVA with Tukey post−test for multiple comparisons. Not significate ns is normally this meaning.

Given that ID1 physically interferes with the interaction between TCF4 and TWIST1, which activates EMT, we investigated whether TGFβ1 increased TCF4 and TWIST1 interactions. When cells were exposed to TGFβ1, an increased TCF4 and TWIST1 interaction as well as a decrease in ID1 were observed (Figure 6A). Stable overexpression of ID1 was found to reverse the decrease in CDH1 and increase in CDH2 upon TGFβ1 treatment (Figure 6B), indicating that ID1 is linked to TGFβ1-induced EMT. To determine the functional impact of TCF4 on EMT gene expression caused by TGFβ1, alteration of EMT markers upon TGFβ1 was measured in TCF4-silenced A549 lung cancer cells. Figure 6C shows that TCF4 knockdown restored the decreased CDH1 and increased CDH2 and SNAI2 expression upon TGFβ1 treatment (Figure 6C). Increased promoter occupancy of TCF4 on CDH1 and CDH2 promoters was observed in the presence of TGFβ1 (Figure 6D). In addition, TCF4 promoter occupancy upon TGFβ1 treatment was significantly decreased in FOXA1 or PGC1α stably overexpressing A549 lung cancer cells (Figure 6D). These results demonstrate that TCF4 controlled by ID1 may act as a critical transcriptional axis during TGFβ1-induced EMT in lung cancer.

## 3. Discussion

Mechanistically, transcriptional cooperation between ERRα and PGC1α promotes lipogenesis, oxidative phosphorylation, and mitochondrial biogenesis in melanoma and breast cancer [8,11,29]. In prostate cancer, ERRα and PGC1α suppress cancer progression and metastasis by activating fatty acid β-oxidation and the citric acid (TCA) cycle [12,13]. However, the mechanistic role of PGC1α in transcriptional circuits that regulate EMT and pro-metastatic gene expression during cancer metastasis is poorly understood, particularly in lung cancer. FOXA1 is an essential transcription factor for the differentiation of the foregut endoderm, including the pancreas, liver, lung alveolar, and prostate luminal ductal epithelia, indicating that FOXA1 is closely associated with embryonic development and epithelial differentiation [26]. Abnormal expression of FOXA1 and FOXA2 has been observed in multiple types of cancers, including prostate, breast, lung, and esophageal cancer [20]. In the current study, using an ID1 and ID2 promoter luciferase assay, we found that PGC1α interacts with FOXA1 transcription factor and activated the transcriptional activity of FOXA1. PGC1α was found to promote both ID1, ID2, and ID3 expression in lung cancer cells, suggesting that PGC1α-mediated expression of ID families might be dependent on the transcription factor that is activated by PGC1α. Specifically, chromatin immunoprecipitation (ChIP) showed that FOXA1 and PGC1α bound to the proximal promoter region (−0.5 kb) of ID1, indicating that PGC1α transcription coactivates FOXA1-mediated target gene expression.

Since ID1 is predominantly expressed in lung cancer among the ID family, we suggest that ID1 serves as an important factor on EMT induced by the loss of FOXA1 and PGC1α [2]. Indeed, we found that loss of FOXA1 and PGC1α decreased E-cadherin and increased N-cadherin and vimentin expression, which was significantly reversed by ectopic ID1 expression in A549 lung cancer cells. These results indicate that ID1 is closely associated with FOXA1-and PGC1α-mediated EMT gene expression. Consistent with our findings, several reports have shown that FOXA1 attenuates EMT in breast and pancreatic cancers [16,20]. The FOXA1-meditated transcriptional network maintains epithelial cell identity by suppressing EMT-related gene expression in human airway epithelial cells [18].

ID1 suppresses the E-box binding protein TCF4 (ITF-2 or E2-2) and TWIST1-mediated EMT gene expression by decoupling TCF4 from TWIST1 [2]. Given that ID1 is transcriptionally regulated by FOXA1, we found that interaction of TCF4 and TWIST1 and its promoter occupancy on the E-box-containing CDH1 and CDH2 promoter region was significantly increased in FOXA1 knockdown cells compared to control cells. In addition, TCF4 knockdown reversed the alteration of expression of EMT genes, such as CDH1, CDH2, VIM, and PTHLH, in FOXA1-silenced A549 cells, indicating that cooperation between TCF4 and TWIST1, which is inhibited by ID1, is required for FOXA1 and PGC1α-mediated EMT.

In the present study, we found that TGFβ1, an essential EMT-promoting factor, suppresses PGC1α and FOXA1 expression in various lung cancer cells. ID1, downstream of PGC1α and FOXA1, was also downregulated in TGFβ1-treated lung cancer cells. Downregulation of ID1 in TGFβ1-treated cells was diminished by ectopic FOXA1 or PGC1α expression, indicating that FOXA1 and PGC1α are critical for ID1 suppression upon TGFβ1 treatment. Ectopic ID1 expression and TCF4 knockdown attenuated TGFβ1-induced EMT gene expression. In addition, promoter occupancy of TCF4 on CDH1 and CDH2 promoter regions increased by TGFβ1 was significantly attenuated by FOXA1 and PGC1α overexpression. These results indicate that FOXA1 and PGC1α act as important components of the transcriptional network for EMT induced by TGFβ1 by regulating ID1-meditated suppression of TCF4 and TWIST1 in lung cancer.

Given that PGC1α is involved in metabolic homeostasis and energy consumption, the functional role of transcriptional complexes containing FOXA1 and PGC1α in energy metabolism could be considered and further investigated in various metabolic tissues. Indeed, FOXA1 was found to activate peroxisomal fatty acid β-oxidation and ketone body synthesis in hepatocytes [30]. Impaired insulin secretion due to decreased ATP production and increased mitochondrial uncoupling protein 2 (UCP2) in FOXA1-deficient islets was previously observed [31]. In addition, increased ID1 expression is observed in brown adipose tissue (BAT) compared to other metabolic tissues, and ID1 is considered a critical factor for adipogenesis and thermogenesis in BAT [32]. Consistent with this, PGC1α regulates glucose metabolism, including glycolysis and gluconeogenesis [6], and ID1-associated c-Myc-mediated aerobic glycolysis and glutaminolysis have been reported in hepatocellular carcinoma cells [33]. Thus, our findings demonstrate that FOXA1 and PGC1α-mediated transcriptional regulation of ID1 expression may involve energy metabolism in metabolic tissues as well as lung cancer metastasis.

## 4. Materials and Methods

### 4.1. Reagents and Antibodies

Recombinant TGFβ1 protein (240-B) was purchased from R&D system (Minneapolis, MN, USA). Anti-Flag (F3165) mouse monoclonal antibody and V5-affinity agarose bead (A7345) were purchased from Sigma Aldrich (St. Louis, MO, USA). Anti-ID1 (sc-133104), β-actin (sc-47778) and β-tubulin (sc-9104) mouse monoclonal antibodies were purchased from Santa Cruz Biotechnology (Dallas, TX, USA). Anti-E-cadherin (610181) and N-cadherin (610921) mouse monoclonal antibodies were obtained from BD Biosciences. Anti-vimentin (CST-5741) rabbit monoclonal antibody was purchased from Cell Signaling Technology (Danvers, MA, USA). Anti-PGC1α (ST1202) and TCF4 (H00006925-M04) mouse monoclonal antibody was purchased from Merck Millipore (Billerica, MA, USA) and Abnova (Taipei, Taiwan). Anti-TWIST1 (ab50887) mouse monoclonal and anti-FOXA1 (ab170933) rabbit monoclonal antibodies were purchased from Abcam (Cambridge, MA, USA). 2′,7′-dichlorofuorescin diacetate (DCF-DA, D68883) was purchased from Sigma Aldrich (St. Louis, MO, USA).

### 4.2. Cell Culture, Plasmids, shRNA, and Generation of Stable Cell Lines

Lung cancer cell lines (A549, H358, H1666 and Calu-1) were obtained from Korean Cell Line Bank (Seoul, Korea) and cultured in 10% fetal bovine serum and 25 mM glucose containing Dulbecco’s modified Eagle’s medium (DMEM) and Rosewell Park Memorial Institute (RPMI)1640. pLX302-FOXA1-V5 (Addgene #70090), pLKO-FOXA1-shRNA#1 (Addgene #70095) and pLKO-FOXA1-shRNA#2 (Addgene #70096) were gifts from William Hahn. pLKO-PGC1α-shRNA (TRCN0000001165 and TRCN0000001166) was purchased from Sigma Aldrich (St. Louis, MO, USA). Human FOXA1 (207490510100) and GFP (C071) adenoviruses were purchased from Applied Biological Materials Inc (Richmond, BC, Canada). pLX304-V5-GFP (EX-EGFP-LX304), pLX304-V5-ID1 (EX-F0699-LX304) and pLX304-V5-PGC1α (EX-A7990-LX304) lentiviral expression vectors were purchased from GeneCopoeia (Rockville, MD, USA). Human TCF4 targeting siRNA (sc-61657) was purchased from Santa Cruz Biotechnology (Dallas, TX, USA). For adenoviral transduction in A549 and H358 lung cancer cells, GFP or FOXA1 expressing adenoviruses (1000 pfu/cell) were treated and incubated for 3 days. Lentiviral particles harboring EGFP, PGC1α, and ID1 transgene were produced in HEK293T cells. Each lentiviral vector was transfected into HEK293T cells with psPAX2 and pMD2G virus packaging and envelop vectors and incubated for 2 days. Cultured medium was filtered through a 0.45 µm filter and 1 volume of Lenti-X concentrator (Takara-Clontech, Saint-Germain-en-Laye, France) was mixed with 3 volumes of clarified cultured medium, and then mixed samples were centrifuged at 1500× *g* for 2 h at 4 °C. After centrifugation, an off-white pellet was dissolved in phosphate-buffered saline (PBS). To generate stable cell lines, lung cancer cells were incubated with lentiviral particles for 48 h, and then infected cells were incubated with 2 µg/mL of puromycin or 10 µg/mL of blasticidin for 4~6 days for clonal selection.

### 4.3. Heat Map Analysis

Previously performed RNA seq (GSE156833) was used for heat-map analysis [2]. Significant genes (*p* < 0.05 and fold change > 1.5) were selected, and formatted Excel file was used as input and visualized by the Multiple Experiment Viewer software (MEV).

### 4.4. Luciferase Assay

Human ID1 (pGL3-ID1-289) and ID2 (pGL3-ID2-352) promoter luciferase vectors were previously described [2]. ID1 and ID2 promoter luciferase vector was transiently transfected into HEK293T cells with Flag-PGC1α and/or pLX302-FOXA1-V5 by using a Polyfect (Qiagen, Hilden, Germany), and then transfected cells were incubated and stabilized for 2 days. Cell lysates were mixed with luciferase assay buffer, and then luciferase activity was measured by using Luminometer (BioTek, Winooski, VT, USA).

### 4.5. RNA Isolation and Quantitative RT-PCR

TRIzol (Invitrogen, Waltham, MA, USA) and high-capacity cDNA reverse transcription kit (Applied Biosystems, Waltham, MA, USA) was used for total RNA isolation and cDNA synthesis. cDNA was used for Quantitative PCR by using SYBR Green PCR Master Mix (Applied Biosystems). The sequences of the PCR primers (5′-3′) were: CCTGTGATGCTTTTGCTGCTCTTG and AAACTATCAAAATCCAGAGAGTCA for PGC1α; GACCGGTGCAATCTTCAAA and TTGACGCCGAGAGCTACAC for CDH1; CCACCTTAAAATCTGCAGGC and GTGCATGAAGGACAGCCTCT for CDH2; ATTCCACTTTGCGTTCAAGG and CTTCAGAGAGAGGAAGCCGA for VIM; TTGTCATGGAGGAGCTGATG and CGGTGTTCCTGCTGAGCTAC for PTHLH; CTACGACATGAACGGCTGTTACTC and CTTGCTCACCTTGCGGTTCT for ID1; GACAGCAAAGCACTGTGTGG and TCAGCACTTAAAAGATTCCGTG for ID2; CTTCCGGCAGGAGAGGTT and AAAGGAGCTTTTGCCACTGA for ID3; TGGTGATACCTAAAGCCTGGAA and CATGTTGCTGGCCAATAAGG for H36B4; TGGGGGGTTTGTCTGGCATA and ATTGGTTTGGGGTTGTCTTTG for FOXA1; CAGTCGGTGTATGCCTTCTCG and GAGGGACGCCACATTCTCG for GPX1; GGAAGCCATCAAACGTGACTT and CCCGTTCCTTATTGAAACCAAGC for SOD2; TTGAGAGTGGTAGGGCTGCT and CCAAAGGAAAATCTGTGGCA from TGFB1; CCCCGGAGGTGATTTCCATC and GGGCGGCATGTCTATTTTGTAAA from TGFB2; GTGATGGCAAGGTCAACATC and CTCGCAGTAGTTGGCATGAT from INHBA.

### 4.6. Chromatin Immunoprecipitation (ChIP)

EZ-ChIP assay kit (Millipore, Burlington, MA, USA) was used for ChIP assay according to the manufacturer instructions and slight modification as described previously [2]. Cells were incubated with 1% formaldehyde for 10 min at room temperature for fixation. Cells were resuspended in ChIP-lysis buffer, and then chromatins were sheared by sonication with an ultrasonic homogenizer (Bandelin Electronic, Berlin, Germany) for four cycles of 5 min (30 s on, 30 s off upon 30% of power). Sheared chromatin samples were diluted in ChIP-dilution buffer. Primary antibodies (anti-TCF4, anti-TWIST1, anti-PGC1α, and anti-FOXA1) were incubated with sheared chromatin samples for overnight at 4 °C. After primary antibody reaction, samples were incubated with salmon sperm DNA blocked protein A/G agarose beads (Millipore, Burlington, MA, USA), and then samples were washed by wash buffer followed by low salt (0.15 M NaCl), high salt (0.5 M NaCl), lithium chloride (0.25 M LiCl) and Tris-EDTA (TE) buffer, respectively. Phenol:chloroform:isoamyl alcohol (25:24:1) was used to isolate immunoprecipitated DNA. Immunoprecipitated DNA was analyzed by quantitative real-time PCR. The sequences of the PCR primers (5′-3′) were: GCTTAGCTTCCTTGCCTCCT and AGCCTCCTGCTCGTCTAGTG for ID1 (−0.5 kb); TGAGACAGGGTCTTGCTTTG and ATGAACCCAAGAAGTGGAGATT for ID1 (−4 kb); CTCCAGCTTGGGTGAAAGAG and GGGCTTTTACACTTGGCTGA for CDH1 (−370~−277); TAGAGGGTCACCGCGTCTAT and TCACAGGTGCTTTGCAGTTC for CDH1 (−169~+32); AGGAGTGGAAGCAGAGCAGT and GGCGTGTAAAGCAGACCATT for CDH2 (+2999~+3112); CTCCACTTCCACCTCCACAT and GAGATCAAGGAGCTGGGGAG for CDH2 (+11,208~+11,227).

### 4.7. Immunoprecipitation and Western Blotting

Cell lysis buffer containing 1% NP-40, 150 mM NaCl, 50 mM Tris-HCl (pH 7.9), 10 mM sodium fluoride (NaF), 0.1 mM EDTA, and protease inhibitor cocktail was used for co-immunoprecipitation. A total of 5 mg of cell lysates were incubated with 1 µg of anti-TWIST1 antibody or 20 µL of V5-affinity agarose bead for overnight at 4 °C. After primary antibody reaction, samples were incubated with 30 µL of protein A/G agarose bead for 4 h at 4 °C. Protein complexes were washed with 1 mL of wash buffer containing 0.5% NP-40, 50 mM Tris-HCl (pH 7.9) and 200 mM NaCl three times. Eluted proteins were subjected into SDS-PAGE, and then separated proteins were transferred onto a PVDF membrane (Milli-pore, Burlington, MA, USA). Separated proteins on PVDF membrane were reacted with primary antibodies (1:1000) in blocking solution containing 5% skim milk and 0.05% Tween-20 overnight at 4 °C. HRP-conjugated secondary antibodies (1:10,000) were incubated for 1 h at room temperature. Different expression of target proteins was visualized using an ECL Prime kit (GE Healthcare, Pittsburgh, PA, USA).

### 4.8. Reactive Oxygen Species (ROS) Analysis

ROS were detected using 2′,7′-dichlorofuorescin diacetate (DCF-DA) according to the manufacturer’s instructions. Cells were cultured into 96-well black plates. When cells reached 70% confluency, cells were washed with PBS, then ROS detection probe DCF-DA (25 µM) was added. DCF-DA treated cells were incubated at 37 °C for 30 min, and then ROS levels were measured at Ex/Em 485/535nm using EnVision Multilabel Plate Reader.

### 4.9. Statistical Analysis

Unpaired Student’s *t*-test for two experimental comparisons and one-way ANOVA with Tukey post-test for multiple comparisons were used for statistical analysis. Data are represented as means ± standard deviations (SD). A *p* value < 0.05 was considered statistically significant.

## 5. Conclusions

Our results provide a mechanistic framework showing that (1) TGFβ1 decreases the partnership of PGC1α and FOXA1, resulting in suppression of ID1, and (2) decreased ID1 leads to EMT-related gene expression through the TCF4 and TWIST1-mediated transcriptional axis in response to TGFβ1. Consequently, this framework may be closely linked to EMT-associated chemoresistance, tumorigenesis, and metastasis in lung cancer.

## Data Availability

Not applicable.

## Supplementary Materials

The following supporting information can be downloaded at: https://www.mdpi.com/article/10.3390/ijms23158247/s1.
