# Supplementary material for: PGC1α Cooperates with FOXA1 to Regulate Epithelial Mesenchymal Transition through the TCF4-TWIST1
Source: Int J Mol Sci. 2022 Jul 26;23(15):8247. doi: 10.3390/ijms23158247 (PMC9332154; doi:10.3390/ijms23158247)
Supplement: Supplementary file 1 [file ijms-23-08247-s001.zip › ijms-1811491-supplementary.pdf]

International Journal of
Molecular Sciences

MDPI

Article

PGC1α Cooperates with FOXA1 to Regulate Epithelial Mesen-chymal Transition Through the TCF4-TWIST1

Xue-Quan Fang [1,2,†], Mingyu Lee [3,†], Woo-Jin Lim [1,2], Seonghoon Lee [1,2], Chang-Hoon Lim [1,2] and Ji-Hong Lim [1,2,4,*]

1 Department of Biomedical Chemistry, College of Biomedical & Health Science, Konkuk University, Chungju 380-701, Chungbuk, Korea; gkrrnjs654852@kku.ac.kr (X.-Q.F.); lwj0908@kku.ac.kr (W.-J.L.); samron7@kku.ac.kr (S.L.); lchoo1196@kku.ac.kr (C.-H.L.); jhlim@kku.ac.kr (J.-H.L.)

2 Department of Applied Life Science, Graduate School, BK21 Program, Konkuk University, Chungju 380-701, Chungbuk, Korea; gkrrnjs654852@kku.ac.kr (X.-Q.F.); lwj0908@kku.ac.kr (W.-J.L.); sam-ron7@kku.ac.kr (S.L.); lchoo1196@kku.ac.kr (C.-H.L.); jhlim@kku.ac.kr (J.-H.L.)

3 Division of Allergy and Clinical Immunology, Department of Medicine and Brigham and Women's Hospital, Harvard Medical School, Boston 02115, MA, USA; leemk08@gmail.com (M.L)

4 Center for Metabolic Diseases, Konkuk University, Chungju 380-701, Chungbuk, Korea; jhlim@kku.ac.kr (J.-H.L.)

* Correspondence: jhlim@kku.ac.kr; Tel.: +82-43-840-3567; Fax: +82-43-840-3929

† These authors contributed equally to this work.

Supplementary materials

Supplementary Figure S1

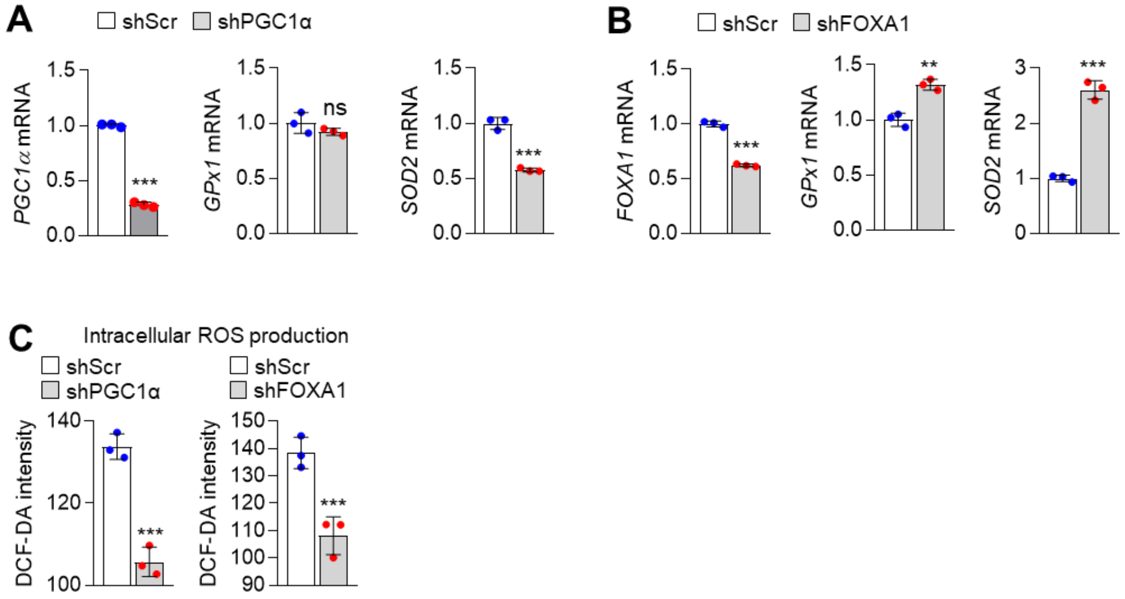

Supplementary Figure S1. The effect of PGC1α and FOXA1 on redox homeostasis. (A and B) GPx1 and SOD2 mRNA expression in (**A**)PGC1α and (**B**) FOXA1 silenced A549 lung cancer cells. (C) Intracellular ROS levels in PGC1α and FOXA1 silenced A549 cells. Values represent mean ± SD (n=3). ** $p < 0.01$ and *** $p < 0.001$ by Unpaired Student's t-test.

Supplementary Figure S2

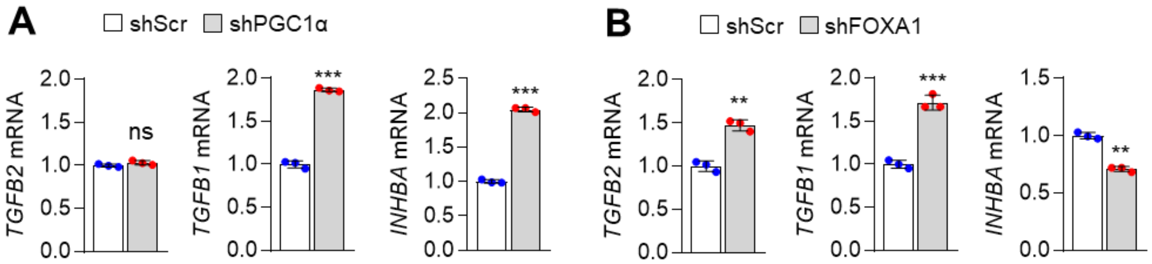

Supplementary Figure S2. Expression of TGFβ1 signaling target genes in PGC1α and FOXA1 silenced cells. (**A** and **B**) TGFB1, TGFB2 and INHBA mRNA expression in (**A**) PGC1α and (**B**) FOXA1 silenced A549 lung cancer cells. Values represent mean ± SD (n=3). ** $p < 0.01$ and *** $p < 0.001$ by Unpaired Student's t-test.
